# Hyperexcitability of the Nucleus Accumbens Is Involved in Noise-Induced Hyperacusis

**DOI:** 10.1155/2020/8814858

**Published:** 2020-11-26

**Authors:** Yuying Liu, Ana''am Alkharabsheh, Wei Sun

**Affiliations:** ^1^Department of Otorhinolaryngology-Head and Neck Surgery, Shanghai General Hospital, Shanghai Jiao Tong University School of Medicine, No. 100 Haining Road, Shanghai, China 200080; ^2^Department of Hearing and Speech Sciences, University of Jordan, Queen Rania Al Abdallah St., Amman, Jordan 11942; ^3^Department of Communicative Disorders and Sciences, Center for Hearing and Deafness, State University of New York at Buffalo, 137 Cary Hall, 3435 Main Street, Buffalo, NY 14214, USA

## Abstract

Reduced tolerance to sound stimuli (hyperacusis) is commonly seen in tinnitus patients. Dysfunction of limbic systems, such as the nucleus accumbens (NAc), may be involved in emotional reactions to the sound stimuli in tinnitus patients. To study the functional changes in the NAc in hyperacusis, we have examined the neural activity changes of the NAc using c-Fos staining in an animal model of hyperacusis. The c-Fos staining was also examined in the medial geniculate nucleus (MGN), a central auditory pathway which has neural projections to the NAc. Postnatal rats (14 days) were exposed to loud noise (115 dB SPL, 4 hours for two consecutive days) to induce hyperacusis (*n* = 4). Rats without noise exposure were used as the controls (*n* = 4). After P35, rats in both groups were put in a behavioral training for sound detection. After they were trained to detect sound stimuli, their reaction time to noise bursts centered at 2 kHz (40-110 dB SPL) was measured. Rats in the noise group showed a significantly shorter reaction time than those in the control group to the noise bursts at high intensities, suggesting the noise exposure induced hyperacusis behavior. The c-Fos expressions in the NAc and the MGNs of the noise group were significantly higher than those of the control group. Our results suggested that early-age noise exposure caused hyperactivity in the NAc and the MGNs which may induce the loudness increase in these rats.

## 1. Introduction

Tinnitus is a phantom sound perception which occurs when there is no external sound in the surrounding environment. Tinnitus patients typically also experience declined sound tolerance or panics to loud sound, known as hyperacusis [1–4]. Patients who suffer from tinnitus and hyperacusis often share the limbic-associated psychological profiles with an increased tendency to anxiety, fatigue, and depression [5–9]. These anxiety disorders can exacerbate the severity of their tinnitus and hyperacusis symptoms [10].

The nucleus accumbens (NAc), a major part of the ventral striatum, is a key structure involved in mediating emotional processing. The NAc receives multiple projections from many brain areas, including the nucleus of the central auditory system, such as the medial geniculate nucleus (MGN) of the thalamus. Brain imaging studies found the structural and functional abnormalities of the NAc in tinnitus patients [11, 12]. Evidence suggested that the NAc can regulate the limbic-auditory interactions and is involved in the occurrence of tinnitus [13]. Recent studies also found that the harshness of tinnitus and hyperacusis is related to the abnormal neural excitability in NAc which causes emotional changes to sound [14–17]. However, how the functional changes in the NAc modulate sound loudness perception is not yet clear.

In our recent studies, we found that early-age hearing loss can cause hyperacusis [18]. Rats with moderate-to-severe hearing loss showed shorter reaction time compared to rats without hearing loss. Physiological studies suggested that hyperexcitability of the central auditory system may be involved in sound behavioral changes [19, 20]. To further understand whether the functional changes of the limbic system are involved in the sound loudness changes, in this study, we used c-Fos immunostaining to detect the neural activity in the NAc and the MGNs in the rats with behavioral evidence of hyperacusis.

## 2. Materials and Methods

### 2.1. Animals and Noise Exposure

Eight neonatal male Sprague-Dawley rats (Harlan Laboratories Inc.) were used in this experiment. They were randomly divided into the control group (*n* = 4) and the noise group (*n* = 4). The care and use of animals were approved by the Institutional Animal Care and Use Committee at State University of New York at Buffalo and conformed to the guidelines issued by the National Institutes of Health.

At postnatal 16 days (P16), rats in the noise group were exposed to a narrow band noise at 115 dB SPL centered at 12 kHz (1 kHz bandwidth) for 4 hours each day in two consecutive days. The sound stimuli were generated by a sound processor (RP2, TDT, Alachua, FL, USA) and presented by a loud speaker (GMI D-49, GMI Sound Crop., NY) placed 10 cm up from the rat's head. The output of the speaker was calibrated by a sound level meter coupled to a half-inch condenser microphone (Model 824 Audiometer, Larson Davis).

### 2.2. Auditory Brainstem Response (ABR) Recording

ABR was used for hearing evaluation for both groups at P35. The hearing test was conducted in a sound attenuation booth, and the rats were anaesthetized with a mixture of ketamine (50 mg/kg) and xylazine (6 mg/kg). Stainless steel needle electrodes (Grass Technologies) were used for the ABR recordings. The noninverting electrode (+) was placed at the vertex, the inverting electrode (-) was inserted near the pinna of the testing ear, and the ground electrode was inserted near the pinna of the contralateral ear. The TDT System 3 (BioSigRP, Tucker-Davis Technology, Alachua, Florida, USA) was used for sound generation and data acquisition. Tone bursts (2 ms duration, 0-100 dB SPL) were used to obtain thresholds at 2, 4, 8, 16, and 32 kHz. The ABR thresholds were obtained by using a step of 5 dB SPL to identify the lowest intensity that elicited a repeatable response.

### 2.3. Sound Detection Training

At P35, rats in both groups were trained for sound detection test using a two-choice operant conditioning task. The detailed behavioral training method was given previously in our published paper [18, 21]. The operant conditioning training apparatus was built using modules from Med Associates Inc. (St. Albans, VT, USA) and was controlled by TDT Hardware (Tucker-Davis Technologies, Alachua, FL, USA) with custom software. The training box had a head entry (nose-poke) used for initiating sound stimuli. Two food dispensers with infrared head-entry detectors were installed on each side of the nose-poke along with a loud speaker on the ceiling of the training box (Fostex FT28D, Tokyo, Japan).

The rats were in food restriction before the behavioral training, and the training was reinforced by the palatable food pellets (Bio-Serv, NJ, USA). During the training, first, they need to initiate a sound stimulus by poking the middle head entry. Then, they need to poke the right food dispenser (H-side) upon perceiving a loud sound (90 dB SPL) and the left food dispenser (L-side) for a soft sound (50 dB SPL). Poking the correct side of the food dispenser was rewarded with food pellets; poking the wrong dispenser, no food pellets were rewarded and they could not start a new trial in 10 seconds. To prevent rats from randomly poking the nose-poke without paying attention to the acoustic stimuli, rats must keep their noses in the head entry for 1 second until a sound was presented. Withdrawing from the nose-poke less than 1 second would not trigger an acoustic stimulation and the food pellet would not be released. The loud and soft sound stimuli were presented in a random order during the training.

After achieving 95% accuracy in the sound detection training, the reaction time to narrow-band noise bursts (50 ms duration, centered at 2 kHz) was tested. Sound was presented at a random order (40-110 dB SPL, 10 dB step), and rats were required to poke the food dispenser within 10 seconds after initiation of sounds. Poking on either side of the food dispenser was rewarded with food pellets. A rat typically earned about 250 pellets during a test typically lasting 40 minutes. The reaction time was defined as the time between the onset time of the acoustic stimulation to the time that the rat withdrew his nose from the nose-poke. Only the trials that led to a reward were used to calculate the reaction time. A training session on the second day of the test was used to reinforce the stable operant performance.

### 2.4. Immunofluorescence Staining

After the behavioral test, all the rats in both groups were used for the c-Fos staining. Rats were placed in a sound attenuating booth for two hours before they were euthanized with carbon dioxide. They were then perfused transcardially with 10% phosphate formaldehyde. Their brains were taken out for postfixation in 10% formaldehyde phosphate for overnight before being transferred to a 30% sucrose in 0.1 M phosphate buffer solution (PBS) for 48 h at 4°C. For each animal, we processed a set of serial sections. Structures were delineated according to anatomical atlases [22]. Sections for the NAc were sampled from bregma 2.04 mm to bregma 1.44 mm, and the MGNs were sampled from bregma −5.76 to −6.24 mm. Coronal serial cryosections were cut to 40 *μ*m thickness on a freezing microtome (HM 505N), and the sections (encompassing the MGN and NAc) were rinsed in 0.1 M PBS.

All the immunostaining processing was performed using free-floating sections. First, the sections were removed from the cryopreservative and rinsed in PBS. Then, the sections were blocked in blocking buffer (5% normal donkey serum, 0.3% Triton X-100 with PBS) for 30 min. The primary antibody, a rabbit anti-c-Fos antibody (diluted 1 : 300; Millipore, Temecula, CA, USA), was added to the sections and incubated overnight at 4°C on a tissue rocker. The sections were then rinsed and incubated with a donkey anti-rabbit secondary antibody labeled with Alexa Fluor 488 (diluted 1 : 300; Abcam, Cambridge, MA, US) for 2 hrs and then incubated with TO-PRO-3 iodide (1 : 500, Thermo Fisher Scientific, Waltham, MA, US) for 20 min at room temperature. The sections were rinsed and mounted on Fisher “Superfrost” polarized slides (Fisher Scientific, Pittsburg, PA, USA), and the images were acquired with a confocal microscope (Zeiss LSM510).

### 2.5. c-Fos-Positive Cell Counting

Images were captured at a 63x magnification oil immersion lens with numerical aperture of 1.4. For quantitative analysis of c-Fos-positive cells, three representative images from each of three serial sections were captured. Each c-Fos-positive nucleus was counted to calculate the average number of the positive staining under double-blind conditions with the ZEN lite software 2012 (Zeiss, Germany).

### 2.6. Statistical Analysis

GraphPad Prism software (GraphPad Software, San Diego, CA) was used for plotting and statistical analysis. Results were presented as mean ± standard error of the mean (SEM). Student's *t*-tests were used for analyzing the results for ABR, reaction time, and c-Fos staining. *P* < 0.05 was taken to be statistically significant.

## 3. Results

### 3.1. ABR Results

**Figure 1:**
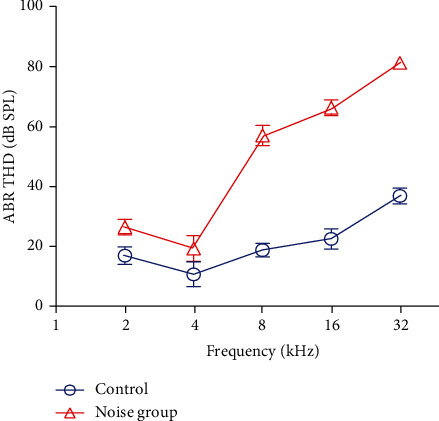
Thresholds of auditory brainstem response (ABR) measured from rats in the control group (*n* = 4) and the noise group (*n* = 4). The mean ABR thresholds of the noise group were significantly higher than those of the control group at 8-32 kHz, not at 2-4 kHz.

ABR thresholds were obtained at P35from the rats in both groups. The mean ABR thresholds of the noise group (*n* = 4) were 40-50 dB higher than those of the control group (*n* = 4). The differences in the ABR threshold of the noise group and the control group were significantly different at 8, 24, and 32 kHz (Student's *t*-test, *P* < 0.05, Figure 1). At 2 and 4 kHz, the ABR thresholds in the noise group had no statistical difference with the control group (Student's *t*-test, *P* > 0.05).

### 3.2. Behavioral Training for Sound Detection Test

**Figure 2:**
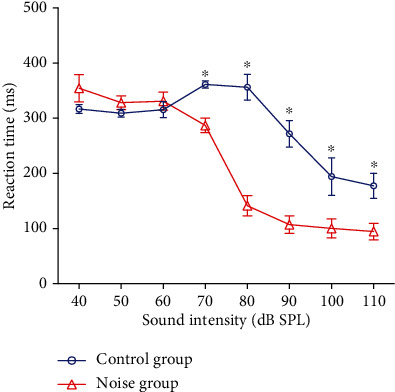
The reaction time-intensity functions measured from the rats in the control group (*n* = 4) and the noise group (*n* = 4) using narrow-band noise centered at 2 kHz (40-110 dB SPL, 10 dB step). At high intensity sound levels (>70 dB SPL), the reaction time in the noise group was significantly shorter than that in the control group (Student's *t*-test, *P* < 0.05), suggesting an increased loudness response.

Rats in the noise group (*n* = 4) and the control group (*n* = 4) underwent operant training for sound detection after P35. A narrow band noise centered at 2 kHz was used for sound detection test to avoid the effect of hearing loss. After 3-4 weeks of training, the accurate detecting rate reached to 95%. Then, the sound reaction time was measured at different intensities (40-110 dB SPL). At the low intensities of sound stimuli (<70 dB SPL), the average reaction time of the noise group was similar to that of the control group. The sound reaction time decreased significantly when sound intensity increased. The sound reaction time of the noise group was significantly shorter than the control group at high intensities (>70 dB SPL, Student's *t*-test. *P* < 0.05, Figure 2). The average reaction time of the noise group (*n* = 4) was 287.1 ± 13.4 ms, 141.3 ± 18.7 ms, 107.1 ± 15.9 ms, 100.3 ± 17.3 ms, and 94.8 ± 14.9 ms at 70, 80, 90, 100, and 110 dB SPL, respectively, whereas the control group (*n* = 4) was 361.3 ± 6.6 ms, 356.1 ± 23.4 ms, 271.8 ± 24.2 ms, 194.3 ± 33.9 ms, and 177.6 ± 22.7 ms at 70, 80, 90, 100, and 110 dB SPL, respectively.

### 3.3. c-Fos Expression in the MGNs and the NAc

**Figure 3:**
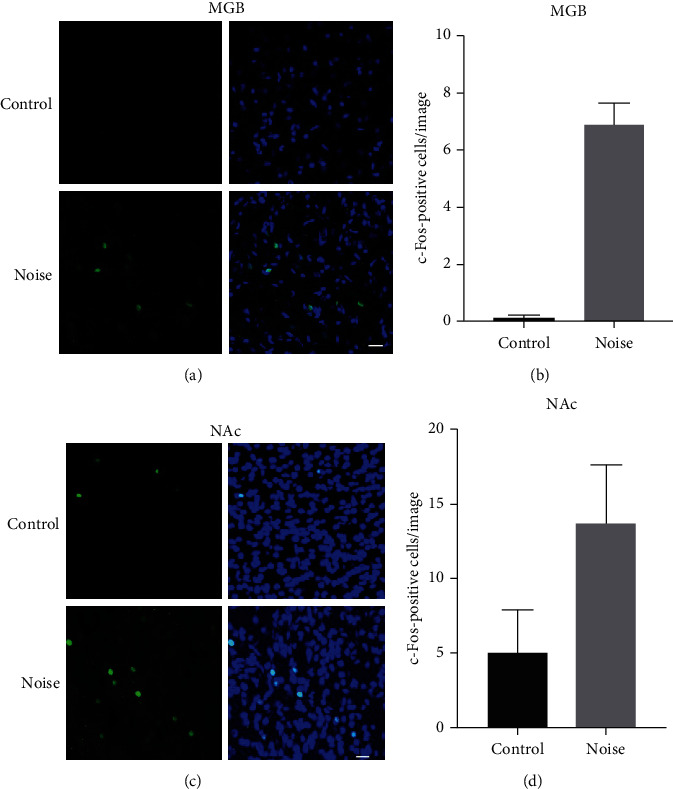
The c-Fos expression detected in the medial geniculate nucleus (MGN) and the nucleus accumbens (NAc) in rats. The nuclei were stained for c-Fos (green) and were visualized with TO-PRO-3 iodide (blue) (63x magnification oil immersion lens with numerical aperture of 1.4). Marker: 10 *μ*m. (a, b) c-Fos has very few expressions in the MGN of the rats in the control group but was significantly expressed in the MGN of rats in the noise group. The c-Fos-positive cell counting showed significant deference (Student's *t*-test, *P* < 0.05). (c, d) c-Fos expression increased obviously in the noise group than in the control group. The number of c-Fos-positive cells of the NAc in the noise group increased significantly compared with that in the control group (Student's *t*-test, *P* < 0.05).

The expression of c-Fos was evaluated in frozen sections of the MGN and the NAc after the behavioral tests. The c-Fos expression in the MGN was very weak in the control group and relatively stronger in the noise group (Figure 3(a)). The difference of the number of c-Fos-positive cells in the two groups was statistically significant (Student's *t*-test, *P* < 0.05). c-Fos expression of the NAc in the noise group was also significantly higher than that in the control group (Figure 3(c)). The number of c-Fos-positive cells in the NAc of the noise group was significantly higher than that of the control group (Student's *t*-test, *P* < 0.05).

## 4. Discussion

In this study, we tested the effects of early-age noise exposure on sound reaction time and c-Fos expression in the NAc and the MGN in rats. We found that rats with hearing loss at an early age showed a shorter reaction time than the controls, suggesting an increase in loudness perception [23, 24]. The results suggest that early-age noise exposure may cause loudness increase at a super-threshold level [18, 20]. The rats with early-age hearing loss may perceive a louder sound perception than rats without hearing loss, consistent with hyperacusis. Our results are consistent with clinical reports that children who experienced a period of sound deprivation during childhood are more susceptible to developing tinnitus and hyperacusis [25, 26].

To detect the neural activity changes of the limbic systems that may contribute to the sound perception changes, the c-Fos stainings in the NAc and the MGN have been evaluated. c-Fos is a well-established marker to identify activated neurons in the autonomous or central nervous systems after multiple stimuli [27–29]. We found significantly upregulated c-Fos expression in the MGN and the NAc in rats with the hyperacusis-like behaviors. Our data suggested that increased excitation in the NAc and the MGN may be related with sound loudness increases after noise exposure.

**Figure 4:**
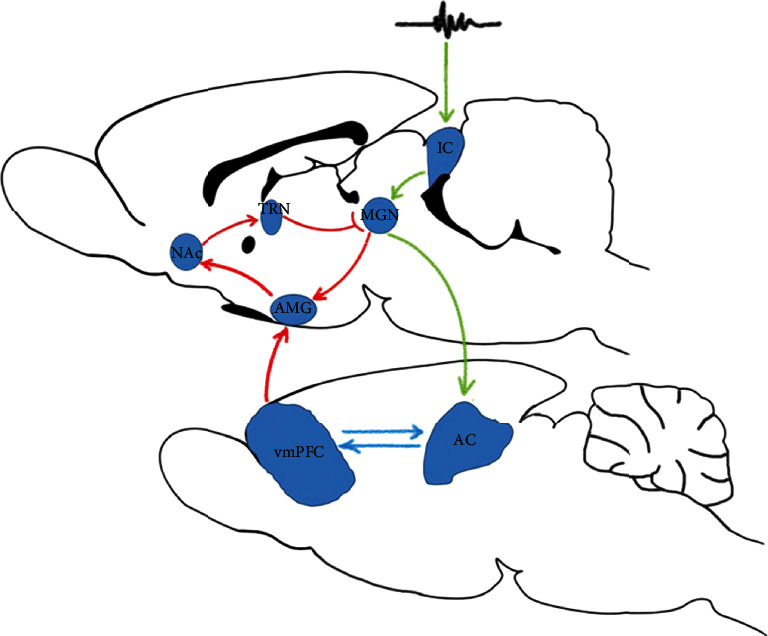
A schematic drawing of the neuronal structures and their projections of the auditory pathway (green lines) and limbic system (red lines) which might be involved in tinnitus and hyperacusis. Please note that the drawing primarily shows the structures and connections most relevant in the context of the proposed mechanism but are not exhaustive. Abbreviations: AC: auditory cortex; vmPFC: ventromedial prefrontal cortex; NAc: nucleus accumbens; AMG: amygdala; IC: inferior colliculi; MGN: medial geniculate nucleus, TRN: thalamic reticular nucleus.

The NAc, which regulates instinctive behavior and emotions, is linked to the auditory system via the MGN. The NAc projects to the MGN via different multisynaptic pathways [30]. Anatomical data indicated that the serotonergic axons from the NAc innervated the thalamic reticular nucleus (TRN) which may have a gain-control function [30, 31]. Electrical stimulation of the NAc produced mostly decreased the neural activity of the MGNs suggesting that the NAc can inhibit the activity of the auditory neurons in the MGN through TRN projections [13, 32, 33] (Figure 4). Rauschecker et al. suggested that the subcallosal areas, such as the NAc, were potentially involved in the cancellation of the tinnitus signal at the thalamic level [34]. They anticipated that tinnitus signals were generated in the auditory system, and failure to be blocked by the limbic system may lead to chronic tinnitus perception. Interestingly, in their study, they also detected hyperactivity in the NAc and the auditory cortex to the sounds at the frequency matched to the patient's tinnitus [35]. Based on their results, we predicted that failure of inhibiting NAc activity may release sound perception in the quiet (causing tinnitus) and exaggerate sound perception in the noise environment (causing hyperacusis). This may explain why tinnitus and hyperacusis are commonly presented together. In our study, increased c-Fos expression of the NAc was found in rats with hyperacusis-like behavior which supports a possible role for the NAc in modulating auditory information in hyperacusis. A recent study also found that injection of 5,7-dihydroxytryptamine (5, 7-DHT), which depleted the serotonergic projection of the NAc to the auditory system, resulted increased acoustical startle response [36]. The study suggested that the serotonergic projection of the NAc may be involved in modulating the neural activity of the MGN in processing sound signals at different intensities.

In summary, the increased neural activity in the NAc and the MGN may be related to the increased loudness perception. A failure on neural modulation between the NAc and the MGN could possibly induce tinnitus and hyperacusis. Our results suggested that early age noise exposure caused hyperactivity of the limbic circuits which may be related to increased loudness perception which is commonly seen in tinnitus and hyperacusis patients [37]. A better understanding of the NAc in hyperacusis may help us to find a novel strategy to reduce tinnitus and hyperacusis.

## Data Availability

Data available on request.
